# Bioinspired multisensory neural network with crossmodal integration and recognition

**DOI:** 10.1038/s41467-021-21404-z

**Published:** 2021-02-18

**Authors:** Hongwei Tan, Yifan Zhou, Quanzheng Tao, Johanna Rosen, Sebastiaan van Dijken

**Affiliations:** 1 NanoSpin, Department of Applied Physics, Aalto University School of Science, P.O. Box 15100, FI-00076 Aalto, Finland; 2 Thin Film Physics, Department of Physics, Chemistry and Biology (IFM), Linköping University, SE-581 83 Linköping, Sweden

**Keywords:** Applied physics, Electronic and spintronic devices

## Abstract

The integration and interaction of vision, touch, hearing, smell, and taste in the human multisensory neural network facilitate high-level cognitive functionalities, such as crossmodal integration, recognition, and imagination for accurate evaluation and comprehensive understanding of the multimodal world. Here, we report a bioinspired multisensory neural network that integrates artificial optic, afferent, auditory, and simulated olfactory and gustatory sensory nerves. With distributed multiple sensors and biomimetic hierarchical architectures, our system can not only sense, process, and memorize multimodal information, but also fuse multisensory data at hardware and software level. Using crossmodal learning, the system is capable of crossmodally recognizing and imagining multimodal information, such as visualizing alphabet letters upon handwritten input, recognizing multimodal visual/smell/taste information or imagining a never-seen picture when hearing its description. Our multisensory neural network provides a promising approach towards robotic sensing and perception.

## Abstract (web summary)

Human-like robotic sensing aims at extracting and processing complicated environmental information via multisensory integration and interaction. Tan et al. report an artificial spiking multisensory neural network that integrates five primary senses and mimics the crossmodal perception of biological brains.

## Introduction

The human multisensory system that integrates the five primary senses, vision, touch, hearing, smell, and taste, as well as their interactions via neural networks in the brain, enables people to explore, learn, and adapt to the world^1–9^. In the human multisensory neural network, sensory receptors (rods and cones, mechanoreceptors, cochlea, smell receptors, taste receptors) convert environmental information into potential changes and encode the potential changes into spike trains with neural spike coding in the cell body. Subsequently, interneurons convey the spike trains from the receptors to the brain’s cerebral cortex, where the information is decoded into sensory perceptions for further processing.

Different from centralized processing in modern computation, which is accurate for repeated tasks and man-made functionalities, distributed processing in biological hierarchical architectures is adaptive and cognitive for efficient analysis of complex multimodal information. Recently, inspired by human sensory processing and perceptual learning, neuromorphic sensing and computing systems with sensors and machine learning algorithms have been demonstrated to sense and process visual^10–12^, tactile^13–18^, auditory^19,20^, and smell and taste information^21,22^, as well as to combine visual and haptic information^18,23^. However, a multisensory system that integrates multiple senses and utilizes crossmodal learning to recognize and imagine multimodal information across different sensory modalities is still absent.

Here, we present a bioinspired spiking multisensory neural network (MSeNN) that integrates artificial vision, touch, hearing, and simulated smell and taste senses with crossmodal learning via artificial neural networks (ANNs). Our MSeNN system senses and converts multimodal physical stimuli to potential changes through various detectors, encodes the potential changes to optical spikes for communication using spike encoders, and decodes, filters, and memorizes environmental information by photomemristors. Finally, ANNs integrate the crossmodal signals with associative learning. The hierarchical and cognitive MSeNN is capable of not only sensing, encoding, transmitting, decoding, filtering, memorizing, and recognizing multimodal information, but it also enables crossmodal recognition and imagination through crossmodal learning for robotic sensing and processing.

## Results

### Artificial MSeNN system with hierarchical processing

**Fig. 1:**
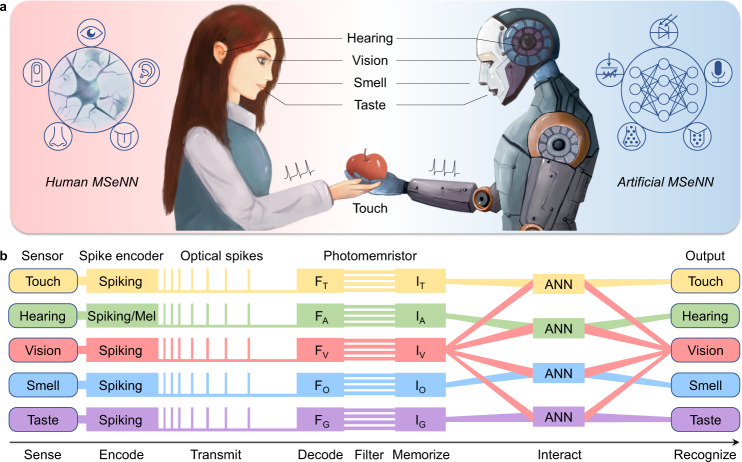
Schematic of the human and artificial MSeNN. **a** Inspired by the five primary sensory systems (vision, touch, hearing, smell, taste) in the human MSeNN and their interaction via neural networks, the artificial MSeNN consists of five artificial sensory systems and their integration via ANNs. **b** Operational diagram of the artificial MSeNN. Sensors (photodetectors, pressure sensors, sound detectors, and simulated smell and taste receptors) convert external stimuli to potentials. Spike encoders encode potentials into optical spikes for communication. The transmitted information is decoded, filtered, and memorized by photomemristors, and the signals are crossmodally integrated and associated by ANNs for crossmodal recognition and imagination.

As the world is multimodal, people learn from and adapt to their environment by sensing, interpreting, and most importantly, associating and learning the crossmodal information they perceive^2,4–9^. Making robotic sensing more human-like requires artificial multisensory systems with high-level cognitive sensing and processing of multimodal environmental information. Figure 1a schematically compares the human and our artificial MSeNN. Both systems consist of five sensory subsystems and neural networks for multisensory data fusion. Inspired by the human distributed and hierarchical sensor networks (Fig. 1b and Supplementary Fig. 1), we fabricated an artificial MSeNN using Si-based photodetectors (vision), MXene-based pressure sensors (touch), and sound detectors (hearing) to convert multimodal information into voltage signals. The olfactory and gustatory receptors (smell and taste) are simulated by nine (etherish, fragrant, sweet, spicy, oily, burnt, sulfurous, rancid, metallic) and five (sweet, sour, salty, bitter, umami) receptor potentials, respectively. The potentials of the five senses are encoded into optical spikes using spike encoders for communication^16^. The conversion to optical spikes avoids voltage degradation and parasitic resistance issues in sensory data communication, and allows accurate encoding with various spike coding principles, including rate coding, temporal coding, or a combination of both. Spike coding is more robust than voltage amplitude coding and it is capable of carrying larger data volumes and distinguishing multiple inputs with a single detector. In our MSeNN system, photomemristors integrate the optical spikes and decode the multisensory information. Each photomemristor works as an artificial optoelectronic (OE) synapse that receives signals from a sensory nerve and produces a post-synaptic current (PSC) at the optical spiking rate (number of spikes per second). Sensory inputs change the spiking rate and PSC of a photomemristor at run-time through a persistent photoconductivity effect, providing built-in memory of sensory information^16^. In the artificial MSeNN, PSC signals representing weighted sensory information are integrated into ANNs to interact with other sensory inputs. Through crossmodal learning, the ANNs construct an associative memory for crossmodal recognition and imagination (Fig. 1b). More details on the individual sensory systems of the artificial MSeNN can be found in the section Methods and Supplementary Note 1.

**Fig. 2:**
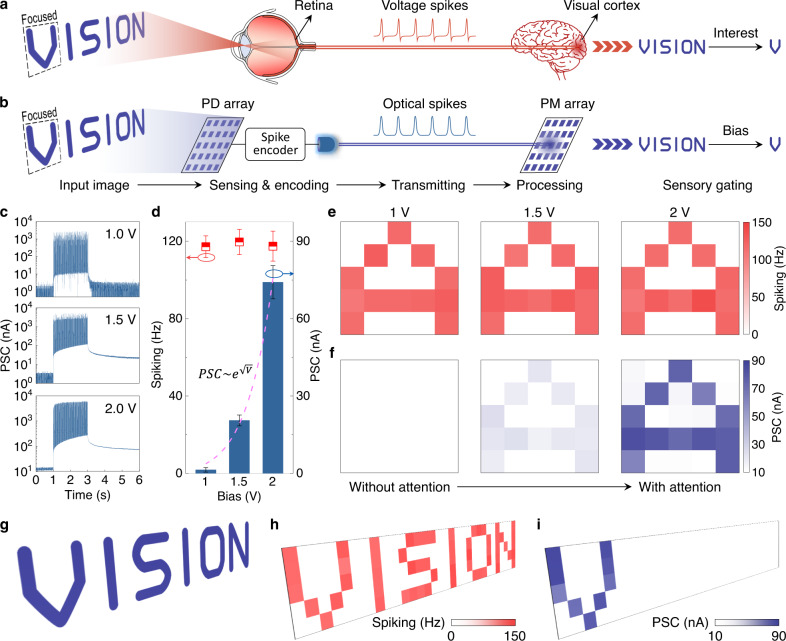
Bioinspired sensory gating in the artificial vision system. **a**, **b** Schematic diagrams of the human and artificial vision systems with attention-dependent information filtering and memory. In the artificial system, built-in memory of visual information detected by photodetectors (PD) is controlled by bias voltages across the photomemristors (PM). **c** PSC signal of a photomemristor in the artificial vision system recorded with different bias voltages (1 V, 1.5 V, 2 V) while the photodetector array is illuminated by the same optical image (letter ‘A’) for 2 s. **d** Spiking rate (number of spikes per second) and PCS value read at *t* = 6 s (3 s after illumination) derived from the signals in (**c**). The dashed line is a fit to the data assuming Schottky emission. The error bars indicate standard deviations in 12 repeated measurements. **e**, **f** PSC spiking-rate and PSC-value maps recorded by a 5 × 5 photomemristor array using different bias voltages (1 V, 1.5 V, 2 V) at *t* = 6 s (3 s after the illumination). The optical input ‘A’ is generated by a blue LED and a shadow mask. **g**–**i** Simulated input image, PSC spiking-rate map, and PSC-value map of the optical input ‘VISION’. Attention is paid only to the first letter of the word ‘VISION’ (2 V bias), whereas all other letters are detected at lower bias voltage (1 V).

Before describing the cognitive functionalities of the artificial MSeNN, we first report on the system’s ability to regulate the built-in memory of sensory information. In biology, a sensory gating effect prevents brain overload by filtering out redundant information (Fig. 2a)^1,3^. Figure 2b illustrates the implementation of sensory gating in the artificial vision system of our MSeNN. In the experiment, a photodetector array, functioning as an electronic retina, detects optically projected letters. Spike encoders encode the sensory information into optical spikes and a 5 × 5 array of photomemristors detect the spike trains. Each photomemristor consists of an indium tin oxide (ITO)/ZnO/Nb-doped SrTiO_3_ (NSTO) Schottky barrier junction. During optical illumination, a persistent photoconductivity effect in the photomemristor produces a PSC signal. The values of the PSC signal vary with the bias voltage across the Schottky barrier (Fig. 2c, d), providing gating-dependent memory of visual information. In contrast, the PSC spiking rate depends only on the sensory input, enabling real-time sensing irrespective of the bias condition. As an example, we demonstrate correct sensing of the optical letter ‘A’ by the photomemristor array using spiking-rate mapping at three bias voltages (Fig. 2e), while the same information is memorized only in the PSC-value map at 2 V (Fig. 2f). Figure 2g–i further illustrates the realization of an attention-dependent memory. Here, ‘attention’ (high bias) is paid only to the first letter of the word ‘VISION’, while the subsequent letters are filtered from the memory by lowering the bias voltage across the photomemristors. All other sensory subsystems of the MSeNN use the same photomemristors as the artificial vision system and, thus, also provide sensory gating capabilities.

### Bioinspired multisensory neuron with crossmodal integration

**Fig. 3:**
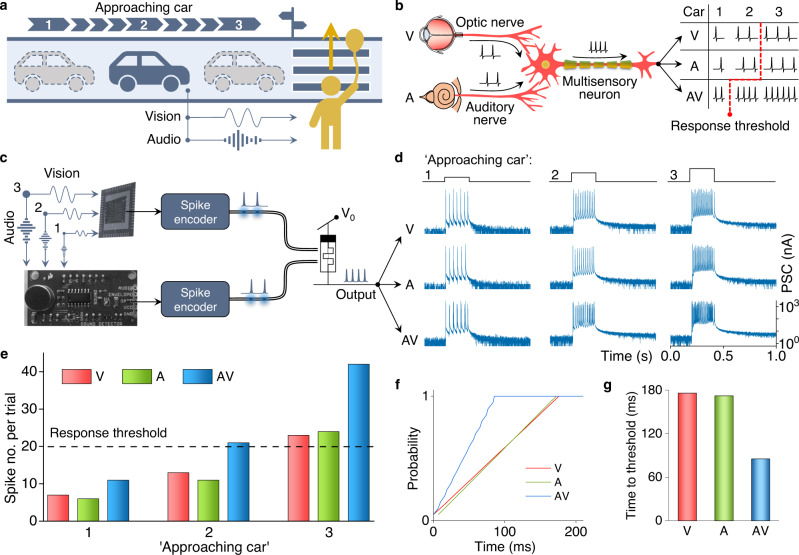
Bioinspired multisensory neuron with crossmodal integration. **a** Simulated situation of a person crossing a road. The person sees and hears an approaching car. The strengths of the auditory and visual input scale with the car’s position, as labeled by numbers 1, 2, and 3. **b** Schematic of a biological multisensory neuron in the superior colliculus. The red dashed line indicates the response threshold of the neuron under visual (V), auditory (A), and combined auditory-visual (AV) stimulation. **c** Artificial multisensory neuron integrating visual and auditory sensory neurons. Three signal levels, weak (1), medium (2), and strong (3), corresponding to the positions of the car, are considered. **d** PSC signal of the photomemristor with rate-coded sensory information during V, A, and AV stimulation. Three signal levels are tested. The sensory input duration is 0.2 s. **e** Number of PSC spikes during each trial in (**d**). We assume a neuronal response threshold of 20 spikes (dashed line). **f**, **g** Response probability (one equals 20 PSC spikes) and latency to reach the response threshold for strong inputs (3).

Multisensory neurons in the midbrain’s superior colliculus directly integrate spikes from different senses to initiate a neuronal response to multimodal environmental events (Supplementary Fig. 2)^24–26^. This cognitive capability raises the awareness and helps people to stay safe. To illustrate this concept with a simple example, we consider a person crossing a road (Fig. 3a). In real life, the person assesses the situation by integrating visual and auditory information, making a well-informed decision on whether to cross the road or not (Fig. 3b). Inspired by this functionality, we implement multisensory neuronal integration by temporally integrating optical spikes from artificial vision and auditory systems using a single photomemristor (Fig. 3c). The vision system acting as the artificial optic nerve consists of a photodetector and a spike encoder with rate coding. The auditory system acting as the artificial auditory nerve comprises a sound detector and a spike encoder with rate coding. As proof-of-principle, we consider weak, medium, and strong sensory inputs (marked by 1, 2, and 3), representing the three car positions in Fig. 3a. Under integrated visual and audio input, the photomemristor produces a larger number of PSC spikes within the actuation period (0.2 s) compared to unisensory activation (Fig. 3d). Here, the spike number under combined audio-visual stimulation is smaller than the sum of spikes recorded during individual audio and visual actuation because of randomly overlapping optical spikes (Fig. 3e). Assuming a neuronal threshold of 20 spikes in our system, Fig. 3e illustrates that the artificial multisensory neuron would detect the car at medium distance (position 2), whereas the input signal needs to be strong (position 3) if vision or sound are used separately. Also, although the PSC spiking threshold number is reached at position 3 for both multi- and unisensory processing, the multisensory neuron reaches the threshold condition more quickly (Fig. 3f,g), triggering a faster response in the case of an emergency. In this example, the visual and audio signals are integrated based on temporal association only, without evaluation of their spatial congruence. Spatial-temporal congruence may be implemented through crossmodal learning before the integration of multisensory signals. Multisensory neuronal integration enables robotic evaluation and action.

### Tactile-visual crossmodal learning and recognition

**Fig. 4:**
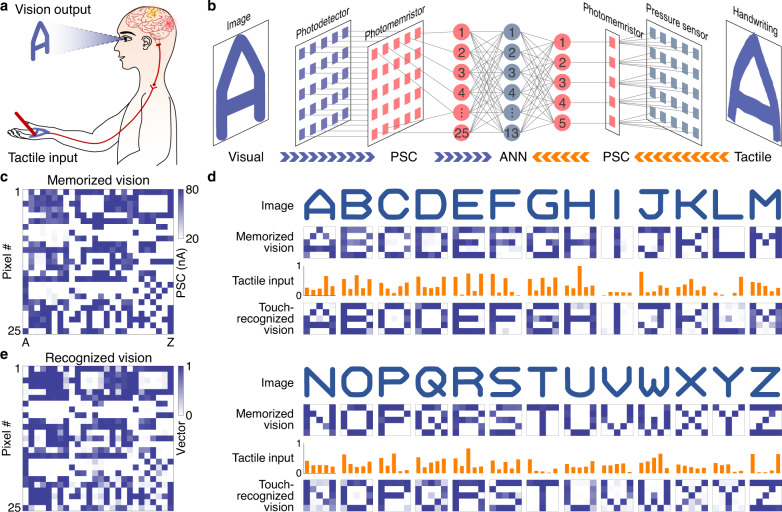
Tactile-visual crossmodal recognition. **a** Illustration of the human ability to recognize and visualize tactile input. **b** Schematic of the artificial tactile-visual system. Tactile inputs from an array of 5 × 5 pressure sensors are dimensionally reduced to five data streams (one photomemristor per five sensors). The visual data stream consists of 25 channels. The ANN consists of five input, thirteen hidden, and 25 output neurons. **c** Vision memory (photomemristors PSC states) recorded after projecting optical images of the alphabet letters A–Z onto an array of 5 × 5 photodetectors for 2 s. The vision memory supervises training of the ANN with tactile inputs. **d** Images, vision memory, and handwritten tactile inputs of alphabet letters A–Z. The fourth and eight rows show the images of alphabet letters that are recognized and reproduced by handwritten inputs after ten training epochs. **e** Summary of reproduced vision vectors. The data correspond closely to the vision memory shown in (**c**), demonstrating tactile-visual sensory integration and crossmodal recognition.

To comprehensively understand the multimodal world, humans utilize crossmodal learning to adaptively connect and associate multimodal information in the high-level cortical areas of the brain for crossmodal recognition and imagination. Inspired by crossmodal learning, we consider the reproduction of simple images triggered by touch in an integrated tactile-vision system (Fig. 4a, b). In the experiments, tactile and vision information from the same event are detected, encoded, transmitted, decoded, filtered, and memorized in their own subsystem and a trained ANN associates the two data streams. As proof-of-principle, we write the letters of the alphabet by hand onto a 5 × 5 pressure sensor array (Supplementary Fig. 3) and process the input signals using five photomemristors (one for each row of five sensors), thus simplifying the analysis of tactile information to five data streams through dimensionality reduction^16^. The spiking proportions of the PSC signals that the five photomemristors produce during handwriting (Supplementary Fig. 3) are used as ANN inputs. Training of the integrated system by tactile input is supervised by the vision memory of the same alphabet letters. The vision memory comprises the PSC states of 25 photomemristors recorded after projecting the optical images of alphabet letters onto an array of 5 × 5 photodetectors for 2 s (Supplementary Fig. 4 shows an example for the letter ‘A’ and statistical analysis of the photomemristors). The vision memory of each alphabet letter is shown in Fig. 4c, and the second and sixth row of Fig. 4d. After training, the tactile-vision system is capable of recognizing handwritten alphabet letters and reproducing their visual image with an accuracy of 92% (Supplementary Fig. 5). The fourth and eighth rows of Fig. 4d depict the A–Z images that the tactile inputs produce when a letter is written without seeing (Fig. 4e shows a 2-dimensional map of the result). Crossmodal learning in the artificial tactile-vision system, inspired by the ability of humans^27^ and animals^28^ to reproduce visual information upon touch, facilitates robotic touch-vision coding, learning, and memory.

### Auditory-visual/olfactory/gustatory crossmodal learning, recognition, and imagination

**Fig. 5:**
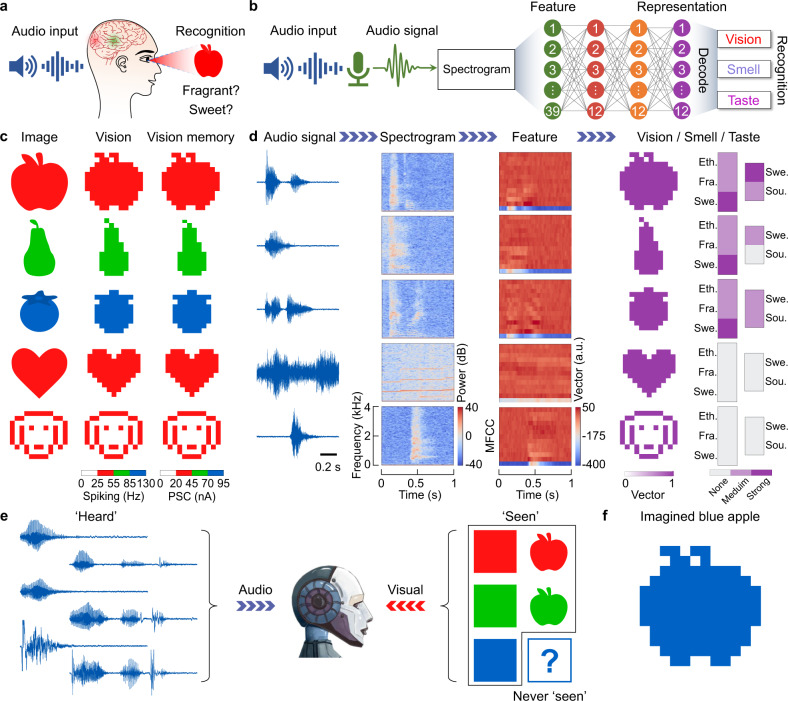
Auditory-visual/smell/taste crossmodal recognition and imagination. **a** Illustration of the human ability to recognize and visualize audio input. **b** Schematic of the artificial auditory-vision/olfactory/gustatory system. Mel spectrograms convert the audio inputs into 13 × 3-dimensional features feeding the ANN. Visual data processed by 12 × 12 photodetectors and photomemristors, together with olfactory and gustatory vectors (Supplementary Fig. 6), are encoded into 12-dimensional features via an autoencoder (Supplementary Fig. 7) to represent the image, smell, and taste information. The ANN consists of 4 layers with 39 input, 12 hidden, 12 hidden, and 12 output neurons (image/smell/taste representation). **c** Detected image (spiking rate of PSC) and vision memory (PSC values after visual input) of an apple, pear, blueberry, heart, and dog. The memorized vision, smell, and taste vectors (Supplementary Fig. 6) are encoded into the representations via the autoencoder to supervise the training of the ANN with audio inputs /ˈapəl/, /pɛː/, /ˈbluːbəri/, music from the song ‘My heart will go on’, and the barking of a dog. **d** Recognized and reproduced image, smell and taste of an apple, pear, blueberry, and the reproduced image of a heart and dog upon associated audio input (spoken words, music, barking). Here, 2200 data sets with different accents (British/Chinese, male/female, child/adult) and two kinds of dog barking (Labrador Retriever and Cocker Spaniel) were divided into two parts, one with 1980 data sets for training and another with 220 data sets for testing. **e** Illustration of supervised training of the auditory-vision system using colors and apples. A blue apple is neither ‘seen’ nor ‘heard’ during the training process. **f** Imagination of a blue apple by the trained system when /bluː, ˈapəl/ is given as audio input after training.

Besides tactile-visual association, humans are also capable of crossmodally reproducing image/smell/taste information when hearing a description of an object^29–31^. Inspired by auditory-visual/olfactory/gustatory sensory interactions and data fusion, we characterize an integrated auditory-vision/olfactory/gustatory system (Fig. 5a, b). In this system, sound detectors pick up the audio input, and Mel spectrograms convert the audio signals into 39-dimensional feature vectors (see Methods). The vision memories of images projected onto a 12 × 12 array of photodetectors (Fig. 5c), and the smell/taste vectors (Supplementary Fig. 6) are automatically converted into 12-dimensional feature vectors by an autoencoder in an unsupervised manner (Supplementary Fig. 7). The learned 12-dimensional representations containing the image/smell/taste information supervise the training of the ANN with audio input. Figure 5d demonstrates the successful reproduction of multisensory data (image/smell/taste) upon hearing. In this example, we trained the auditory-vision/olfactory/gustatory system to produce the representations with encoded image, smell, and taste information when hearing the words ‘apple’, ‘pear’, and ‘blueberry’ pronounced by people (male, female, child) with different British and Chinese accents. We also played the song ‘My heart will go on’ from the movie ‘Titanic’ and associated it with an image of a heart. Additionally, barking by a Labrador Retriever and Cocker Spaniel is associated with an image of a dog. During training, we used 1980 sets of audio signals, including ‘apple’, ‘pear’, ‘blueberry’ with random accents, music fragments, and dog barking, as input under the supervision of the learned representations (Supplementary Fig. 7) with encoded image/smell/taste information of the ‘apple’, ‘pear’, ‘blueberry’, ‘heart’, and ‘dog’. The test results depicted in Fig. 5d are obtained after learning and decoding, demonstrating the potential of overall semantic recognition. The accuracy and loss of the auditory-vision/olfactory/gustatory system during training and testing are plotted in Supplementary Fig. 8. Here, accuracy is defined as the recognition rate of the 12-dimensional representations learned by the autoencoder in each epoch. Loss is defined by the mean square error function (Supplementary Note 1). Random test results demonstrate successful audio-visual recognition for different accents and two dog-barking sounds (Supplementary Fig. 9a). Additionally, to assess whether the network recognizes audio inputs not used during training, we trained another network using several accents and tested the system with a completely new accent. The results shown in Supplementary Fig. 9b demonstrate that high recognition accuracy is attained irrespective of the accent used during training and testing.

In the previous demonstrations, the recognized images have been ‘seen’ before during autoencoding and ANN training. However, based on past experience and memory, the human brain can also imagine the picture of an object that was never seen before, or even does not exist in reality, when hearing its description. This crossmodal sensory imagination capability allows people to speculate about environmental information and to create new concepts or objects with only limited knowledge^4,32^. Inspired by this higher level cognitive functionality, we train our artificial auditory-vision system to imagine the picture of an object upon audio input. As demonstrator, we consider a blue apple. After learning the colors ‘red’, ‘green’, and ‘blue’, as well as the fruits ‘red apple’ and ‘green apple’ (Fig. 5e), the auditory-vision system is able to imagine the picture representation of a blue apple when hearing /bluː, ˈapəl/ without ever hearing or seeing it (Fig. 5f and Supplementary Fig. 10). Artificial imagination in our MSeNN offers cognitive flexibility and holds the potential of self-learning and robotic creativity. Finally, Supplementary Fig. 11 summarizes the interactivity between the five primary senses in our artificial MSeNN.

## Discussion

We presented an artificial MSeNN system that integrates five artificial senses with multimodal sensing, spike encoding, memristive processing, and crossmodal recognition. The MSeNN uses multiple sensors to sense, spikes to encode sensory information, and arrays of photomemristors to interpret, filter, integrate, and memorize multisensory information at hardware level. The built-in memory and information filtering properties of photomemristor arrays facilitate supervised training of an ANN, which provides associations between the five senses, enabling high-level cognitive capabilities including crossmodal recognition of vision/smell/taste information upon tactile or audio input, and crossmodal imagination of a never-before-seen picture when hearing its description. Although the demonstrations are simple compared to biological systems, the hierarchical architectures, principle concepts, and cognitive functionalities of our MSeNN system allow for straightforward extensions to other sensory integrations, providing a promising strategy toward robotic sensing and cognition.

While the concepts and functionalities of the artificial MSeNN with five integrated senses provide a fundamental and essential step toward robotic sensing and perception, practical applications require more human-like sensors. For instance, the modular structure of the MSeNN allows the integration of visual sensors that behave like biological retinas^10^ or tactile gloves with a high density of pressure sensors^14^. The larger data streams that such sensors produce demand more photomemristors and an extended ANN. Both the hardware and software of the MSeNN can be scaled for such tasks. Moreover, dimensionality reduction, as demonstrated in the tactile-vision system and the use of an autoencoder, effectively condenses the sensory information. Besides improvements on the sensor side, it would also be interesting to explore other coding schemes in future works. Compared to recent artificial multisensory systems (see Supplementary Table 1), the spiking rate and temporal coding principles employed in this work allow for flexible processing of sensory information (e.g., sensory gating, dimensionality reduction) and crossmodal learning via ANNs. It is also noteworthy that, based on this research, complex overall semantic recognition for robotics could be realized by using more complex multimodal autoencoders^33^ for the encoding of large volumes of multisensory data.

## Methods

### Artificial vision system

The artificial vision system consists of a silicon-based photodetector array, spike encoders, and photomemristors (Supplementary Fig. 1b). The photodetector array is made of Au/Si junctions bonded to a PCB board with Wire Bonder Delvotec 53XX. The Au/Si junctions were fabricated by atomic layer deposition (ALD), photolithography, etching, and magnetron sputtering. The photodetector array has a pixel size of 100 μm × 100 μm. In the experiments, we used 5 × 5 pixels for the detection of alphabet letters (Fig. 4) and 12 × 12 pixels for the imaging of apples, pears, blueberries, hearts, and dogs (Fig. 5). The spike encoders consist of a commercial ring oscillator, edge detector, amplifier, and light-emitting diode (LED). The ring oscillator uses three NOT gates to form an oscillating signal. The frequency of the oscillation scales with the amplitude of the input signal, enabling biomimetic rate coding of sensory information. The edge detector consists of two NOT gates, one AND gate, one resistor, and one capacitor. It detects the edge of the signal and generates voltage spikes with a fixed width of 1 ms. The amplifier is used to adjust the amplitude of the spikes to the working voltage of the LED. The LED produces 1 ms optical spikes with encoded sensory information^16^. Photomemristors detect and memorize the information encoded in the optical signals. The photomemristor array is made of ITO/ZnO/NSTO junctions fabricated by ALD, photolithography, etching, and magnetron sputtering. Conductive NSTO substrates function as the bottom electrode of the photomemristors. To form a Schottky barrier, photosensitive ZnO films with a thickness of 60 nm were deposited by magnetron sputtering (5.8 × 10^−3^ mbar, Ar 16 sccm, O 4 sccm, power 60 W) on top of the NSTO substrates. Transparent and conductive ITO top electrodes were grown by magnetron sputtering (3.4 × 10^−3^ mbar, Ar 10 sccm, power 50 W) through a metal shadow mask. The photomemristors have a working area of 100 µm × 100 µm. More information about the optoelectronic properties of the photomemristors are given in Fig. 2 and Tan et al.^16^.

The performance of the artificial vision system was characterized by Keithley 2400, Keithley 4200, and Agilent B1500 instruments. The input images were projected onto the photodetector array using red/green/blue LEDs and shadow masks. PSC signals of the photomemristors were recorded with an Agilent B1500 semiconductor device parameter analyzer while projecting optical images onto the photodetector array for 2 s. During image detection, the visual input is firstly converted into potential changes, then encoded to optical spike trains by the spike encoders, and finally decoded in the form of the PCS spiking rate, and if required, memorized by the photomemristors in the form of the PSC state. Operation of the artificial vision system is demonstrated in Fig. 2, which illustrates the system’s ability to filter information through sensory gating.

### Artificial tactile system

The artificial tactile system consists of pressure sensors, spike encoders, and photomemristors (Supplementary Fig. 1b). The pressure sensor array is made of MXene on flexible substrates. MXene is a 2-dimensional metal carbide/nitride^34,35^ exhibiting conductivity changes in response to external pressure^36–39^. For its derivation, we prepared an etchant by adding 0.8 g of LiF to 10 mL of 9 M HCl and left it under continuous stirring for 5 min. A total of 0.5 g of Ti_3_AlC_2_ powder (450 mesh) was gradually added (over the course of 5 min) to the etchant, and the reaction was allowed to run for 24 h at room temperature. The acidic mixture was washed with deionized H_2_O first via centrifugation (1 min per cycle at 1860 *g*) for 2 cycles. After each cycle, the acidic supernatant was decanted as waste followed by the addition of fresh deionized H_2_O before another centrifuging cycle. Then 3 M HCl and 1 M LiCl were used for additional washing via centrifugation (each for 3 cycles, 1 min per cycle at 1860 *g*). Finally, the mixture was washed with deionized H_2_O for another 2 cycles. These washing cycles were repeated until a pH of 4−5 was reached. The final sediments were re-dispersed in deionized H_2_O (0.2 g MXene per 50 mL of water), deaerated with N_2_, followed by sonication for 20 min. The mixture was then centrifuged for 30 min at 1046 *g*, and the supernatant was collected.

The pressure sensors were fabricated by patterning Au/Ta electrodes with a thickness of 50 nm/5 nm on flexible substrates using magnetron sputtering (Ta: DC 30 W, Ar 30 sccm, 25 s. Au: DC 30 W, Ar 30 sccm, 300 s). In parallel, MXene was transferred onto PDMS layers. Before attaching the MXene, the surface of the PDMS layer was made hydrophilic by a plasma treatment (1 min). Then, the MXene solution was dropped on a selected area of the PDMS layer and the solution was evaporated in air. Finally, the PDMS layer with MXene was aligned and mounted onto the flexible substrate with metal electrodes. Supplementary Fig. 3a illustrates the structure of the MXene-based pressure sensors.

The spike encoders and photomemristors of the artificial tactile system are identical to those used in the artificial vision system (see previous section). In the artificial tactile system, a 5 × 5 pressure sensor array detects handwritten letters and converts the information to voltage signals (experiments shown in Fig. 4). The 25 voltage signals are encoded to optical spikes by five spike encoders (each row of five pressure sensors in the array connects to one spike encoder). The hardware of the artificial tactile system thus reduces the dimensionality from 25 to 5 (see Tan et al.^16^ for details), simplifying recognition and subsequent data analysis. The optical spikes with encoded handwritten information are decoded to 5-dimensional spiking proportions by five photomemristors (Supplementary Fig. 3b, c). The information is memorized through weight changes (PSC states of the photomemristors).

### Artificial auditory system

The artificial auditory system uses commercial SparkFun sound detectors. The sound detectors convert spoken words, music, and dog barking to electrical wave signals. In the artificial system emulating multisensory neuronal integration (Fig. 3), the potentials of a photodetector (vision) and a sound detector are encoded into optical spikes using spike encoders. A single photomemristor integrates and detects the optical spikes. The number of PSC spikes produced by the photomemristor depends on the senses used (V, A, or AV) and the strength of the input signals. In the proof-of-concept experiments, three input levels are tested. The input levels corresponded to three positions of an approaching car as illustrated in Fig. 3a. Multisensory information integration at hardware level, as demonstrated in our artificial multisensory neuron system, facilitates faster and better-informed decision making (Fig. 3e–g).

For crossmodal learning in the auditory-vision/olfactory/gustatory system (Fig. 5), we used Mel spectrograms to represent the electrical wave signals of sound detectors before conveying the audio inputs to the ANN. Mel spectrograms with an emphasis on audible frequencies are a standard tool for the processing of sound in speech recognition. In our artificial auditory system, a Mel-weighted filter bank is applied to the input signal to produce Mel spectrograms. After generating the Mel spectrograms, they are represented by 13 × 3-dimensional vectors, containing temporal and frequency information of the detected sound. The 39-dimensional vector features are used for further processing in the ANN. To realize crossmodal recognition and imagination of the image/smell/taste upon hearing (Fig. 5), the ANN integrates sound signals (Mel spectrogram data) and representations learned via an autoencoder. In the experiments, we recorded the spoken words ‘apple’, ‘pear’, and ‘blueberry’ using people with different accents (British and Chinese), genders (male and female), and ages (child and adult). Each spoken word corresponding to one image was recorded for about 200 times. Additionally, a fragment of the song ‘My heart will go on’ from the movie ‘Titanic’ and the barking of a Labrador Retriever and a Cocker Spaniel were used as audio input. Learned representations with encoded image/smell/taste information of the ‘apple’, ‘pear’, ‘blueberry’, ‘heart’, and ‘dog’ (Supplementary Fig. 7b) were used as supervisors for crossmodal recognition (Fig. 5c, d). ‘Red’, ‘green’, ‘blue’, ‘red apple’, and ‘green apple’ sound signals were used for crossmodal imagination of a blue apple (Fig. 5e, f).

### Artificial olfactory and gustatory systems

In the artificial olfactory and gustatory systems, smell and taste senses are simulated by nine (etherish, fragrant, sweet, spicy, oily, burnt, sulfurous, rancid, metallic) and five (sweet, sour, salty, bitter, umami) receptor potentials, respectively^40^. Spike encoders convert the simulated voltages into optical spikes and photomemristors decode and memorize the sensory information (Supplementary Fig. 1). The spike encoders and photomemristors are identical to those used in the artificial vision, tactile, and auditory systems.

### Artificial neural networks

The ANNs connecting multiple senses consist of three or four layers, an input layer, one or two hidden layers (two hidden layers are used in the auditory-vision/olfactory/gustatory system to generate better representations), and an output layer. During training, the input features of an ANN correspond to information detected by one artificial sense and the output features correspond to the information perceived by one or more artificial senses. After training, a new input to the first sense (e.g., a touch or sound) produces a related output of other senses (e.g., an image, smell, or taste). The output is a recognized or imagined representation enabled by crossmodal learning and associative memory. The ANNs used in this work were built in Matlab R2019b and Python. More information on specific ANNs can be found in Supplementary Note 1.

### Autoencoder

We used an autoencoder (Supplementary Fig. 7a) to learn and automatically find the representation of multisensory information (vision, smell, taste). The autoencoder has 154 input neurons, 32 hidden neurons with ReLU activation function, and 12 representation output neurons with ReLU activation function at the encoder side, and 12 representation input neurons, 32 hidden neurons with ReLU activation function, and 154 decoded output neurons with sigmoid activation function at the decoder side. The loss function is a mean squared error function. As input, we used 1000 data sets (1000 combinations of image, smell, and taste vectors) for each object (apple, pear, blueberry, heart, dog) with 10% Gaussian noise, totaling 5000 data sets for all the objects. After 200 training epochs (batch size: 50), 12-dimensional representations with encoded vision/smell/taste information of each object are learned and automatically found (Supplementary Fig. 7b).

## Supplementary information

Supplementary Information.

## Data Availability

The source data underlying the figures in the main manuscript and Supplementary Information are provided as Source Data file. All other data that support the findings of this study are available from the corresponding authors upon reasonable request. Source data are provided with this paper.

## Source data

Source Data

## Code availability

The codes used in this study are available from the corresponding authors upon reasonable request.
