# Supplementary material for: Bioinspired multisensory neural network with crossmodal integration and recognition
Source: Nat Commun. 2021 Feb 18;12:1120. doi: 10.1038/s41467-021-21404-z (PMC7893014; doi:10.1038/s41467-021-21404-z)
Supplement: Supplementary file 1 — Supplementary Information. [file 41467_2021_21404_MOESM1_ESM.pdf]

Supplementary Information

Bioinspired multisensory neural network with crossmodal integration and recognition

H. Tan*, Y. Zhou, Q. Tao, J. Rosen, S. van Dijken*

*Correspondence to: hongwei.tan@aalto.fi (H. T.); sebastiaan.van.dijken@aalto.fi (S. v. D.)

Supplementary Note 1

ANN connecting touch and vision: The ANN that connects the artificial tactile and vision systems has 3 fully connected layers (5 input neurons, 13 hidden neurons, and 25 output neurons (Figure 4)) with backpropagation and mean squared error loss function. The input corresponds to 5-dimensional tactile features (5 PSC spiking proportions, each of which was generated by signals from a row of five MXene pressure sensors) and the output corresponds to the 25 PSC values produced by 25 photomemristors connecting to 25 photodetectors. Therefore, 26 pairs of 5-dimensional tactile vectors and 25-dimensional vision vectors represent the 26 alphabet letters A-Z. All the layers of the ANN are fully connected, i.e., every neuron in one layer connects to all the neurons in the neighboring layer(s). The connection weights are updated during training. The activating function of the hidden layer is a log-sigmoid function, while the output layer uses a tanh function. In the experiments shown in Figure 4, we trained the ANN using 504 sets of tactile vectors of handwritten alphabet letters under the supervision of vision vectors and tested the ANN with 26 sets of tactile vectors. The PSC states of the vision vectors were converted to binary 0 and 1 values by a step function to simplify the recognition process. After training, the alphabet letters were written again by hand onto the 5 × 5 array of pressure sensors. Input of the 26 sets of tactile vectors to the trained ANN produces an output corresponding to the images of the handwritten alphabet letters (touch-reproduced vision). Although the ideal ANN output would correspond to binary 0 and 1 values, images of all alphabet letters (except ‘C’ and ‘D’) are correctly reproduced after only 10 training epochs (Supplementary Fig. 5), demonstrating successful crossmodal visual recognition of handwritten information.

ANN connecting hearing and vision/smell/taste: The ANN that connects the artificial auditory system and the vectors representing the visual/olfactory/gustatory systems has 39 input neurons, 2 layers with

12 hidden neurons, and 12 output neurons (Figure 5b). The input corresponds to 39-dimensional features derived from Mel spectrograms (auditory system) and the output corresponds to a 12-dimensional feature representation that can be decoded to an image, smell, and taste via the decoder part of the autoencoder (see Methods in main manuscript). All the layers of the ANN are fully connected, i.e., every neuron in one layer connects to all the neurons in the neighboring layer(s). The connection weights are updated during training with backpropagation. The activating function of the hidden layer is a ReLU, while the output layer uses a linear function as the representation value is random and larger than one. The loss function is a mean square error function:

$$\mathrm{Loss} = \frac{1}{n}\sum_{\mathrm{i}=1}^{\mathrm{n}}(\mathrm{Y}_{\mathrm{i}}^{\mathrm{Pred}} - \mathrm{Y}_{\mathrm{i}}^{\mathrm{True}})^2$$

where n is the number of samples and Y is the output. For crossmodal recognition of three fruits, a heart, and a dog (Figure 5c,d), the ANN was trained by 1980 sets of 39-dimensional Mel feature vectors under the supervision of 5 sets of 12-dimensional representation vectors. After training, we tested the system using 220 sets of 39-dimensional Mel feature vectors extracted from audio inputs (spoken words, a music fragment, or dog-barking). Figure 5d and Supplementary Figs. 8 and 9 summarize the test results.

ANN connecting hearing and vision for imagination: The ANN used for crossmodal imagination of a blue apple has 3 fully connected layers (39 input neurons, 16 hidden neurons with ReLU activation function, and 4 output neurons with sigmoid function). The loss function is a mean square error function. The ANN with one-hot encoding, using binary 0/1 to represent categories (apple, red, green, blue), was trained by 1242 sets of 39-dimensional Mel feature vectors under the supervision of 5 sets of 4-dimensional binary feature vectors (corresponding to apple, red, green, blue). After 1000 training epochs (batch size: 32, i.e., 32 training examples in one forward/backward pass), the system was tested by 20

sets of 39-dimensional Mel feature vectors corresponding to audio inputs of the spoken word ‘blue apple’ that was never seen or heard. Figure 5e,f and Supplementary Fig. 10 depict the predicted images.

Supplementary Figures

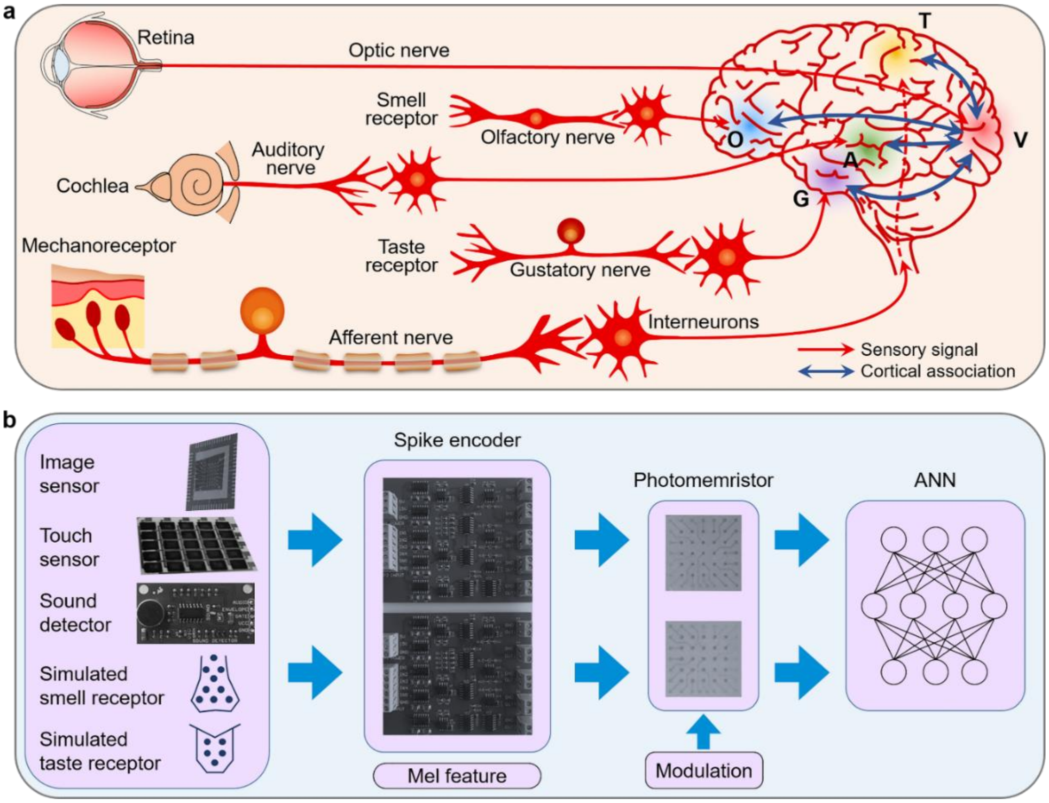

Supplementary Figure 1. Human and artificial MSeNN system. a, Schematic of sensory information transmission from receptors to cortical areas in the brain. The human MSeNN system detects environmental information through various primary receptors, including photoreceptor cells in the retina, mechanoreceptors in the skin, cochlea in the ears, and smell and taste receptors in nose and tongue. The nerve system conveys the encoded sensory information to cortices in the brain as spike trains. Cortical areas of the brain decode, filter and, if necessary, memorize this information. Association of sensory information from different receptors occurs in various parts of the cerebral cortex. In these association areas, the visual, auditory, somatosensory, gustatory, and olfactory impulses are integrated and processed with multisensory fusion. Crossmodal processing of sensory information in the human brain improves the perception of environmental objects[1-6]. **b**, Components of the artificial MSeNN. The system consists of multiple sensors (image sensor, touch sensor, sound detector, simulated smell receptor, and simulated

taste receptor), spike encoders, arrays of photomemristors, and an artificial neural network (ANN). Our artificial sensory system utilizes various sensors to detect environmental information. Signals from silicon-based photodetectors (vision), MXene-based pressure sensors (touch), sound detectors (hearing), and simulated smell and taste receptors are encoded as optical spikes. Arrays of photomemristors integrate, decode, and filter the multisensory information and, if necessary, memorize the input through an increase of their synaptic weight (PSC value of the photomemristor). Crossmodal sensory information is integrated in an artificial neural network, enabling cognitive functionalities such as crossmodal learning, recognition, and imagination. The sensor potentials are encoded into optical spikes by spike encoders[7]. The spike encoders consist of a commercial ring oscillator, edge detector, amplifier, and light-emitting diode (LED)[8,9]. The ring oscillator uses three NOT gates to form an oscillating signal. The frequency of the oscillation scales with the amplitude of the input signal, enabling biomimetic rate coding of sensory information. The edge detector consists of two NOT gates, one AND gate, one resistor, and one capacitor. It detects the edge of the signal and generates voltage spikes with a fixed width of 1 ms. The amplifier is used to adjust the amplitude of the spikes to the working voltage of the LED. The LED produces 1 ms optical spikes with encoded sensory information. Photomemristors detect and memorize the encoded information for further processing.

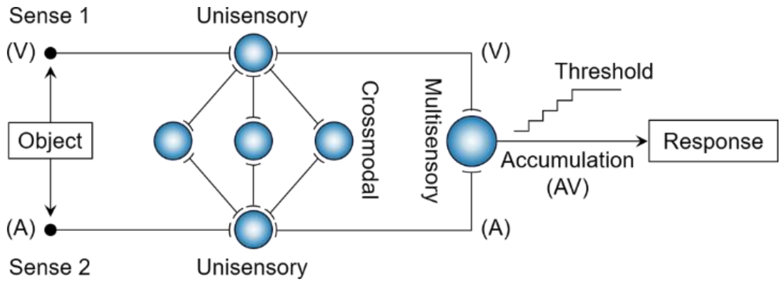

Supplementary Figure 2. Bioinspired multisensory neuron system. The artificial auditory-visual multisensory neuron integrates and processes multisensory information in two different ways. Neuronal multisensory integration requires simultaneous processing of visual and auditory data streams using the same photomemristor (right side of schematic and Figure 3). In the artificial multisensory neuron system, a photodetector and sound detector pick up the optical and audio signals, spike encoders convert the output voltages of these detectors into optical spikes, and a single photomemristor integrates and processes the multisensory information. Simultaneous activation of both senses produces a higher photomemristor PSC spiking rate, enabling faster and more accurate responses in robotic applications. Multisensory association and crossmodal recognition/imagination that involve supervised training of an ANN to connect multiple senses is demonstrated in Figures 4 and 5 of the main manuscript.

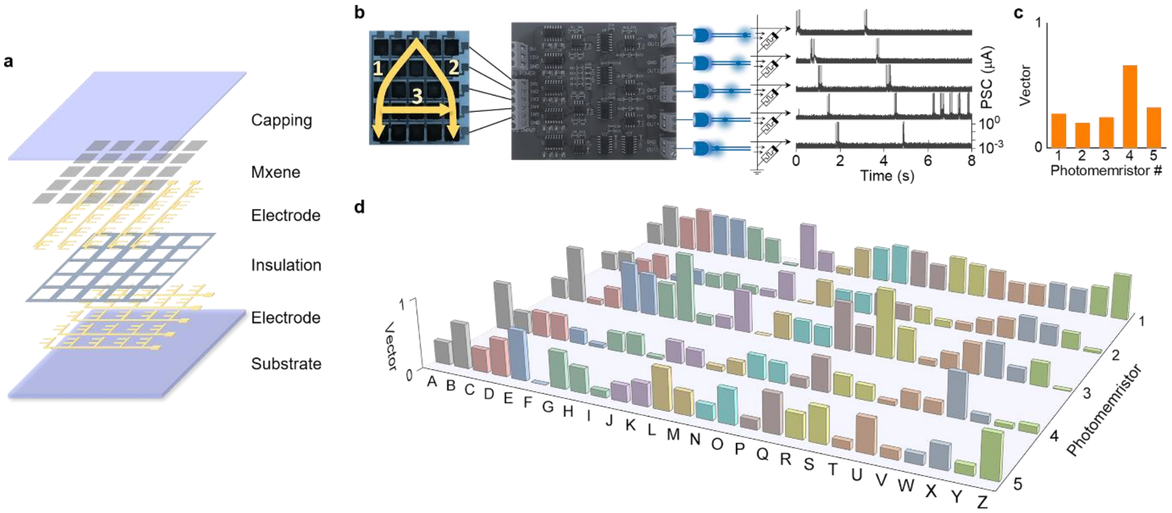

Supplementary Figure 3. Artificial tactile system. a, Schematic of the structure of the MXene-based pressure sensor array. **b**, Schematic illustration of how a handwritten letter 'A' on a 5 × 5 array of pressure sensors is processed in the artificial tactile system. Spike encoders transform the potentials of each row of pressure sensors into optical spikes. Five photomemristors decode and memorize the tactile information through a persistent photoconductivity effect. Dimensionality reduction of sensory signals from 25 to 5 simplifies the recognition process. The photomemristors produce PSC spikes upon pressure input (right panel in (b)). Spiking proportions, defined as $P_i = t_{\text{spiking}}/t_{\text{writing}}$ with t_{spiking} the total spiking time of a photomemristor i recorded in real time and t_{writing} the time it takes to handwrite an alphabet letter, are used as 5-dimensional feature vector. The spiking proportions are not affected by the decaying PSC. The values of the tactile vectors are normalized (0-1) to the maximum spiking proportion in the alphabet A-Z. **c**, Normalized spiking proportion obtained while handwriting the letter 'A' on the pressure sensor array of the artificial tactile system. **d**, Feature dictionary of handwritten letters of the alphabet.

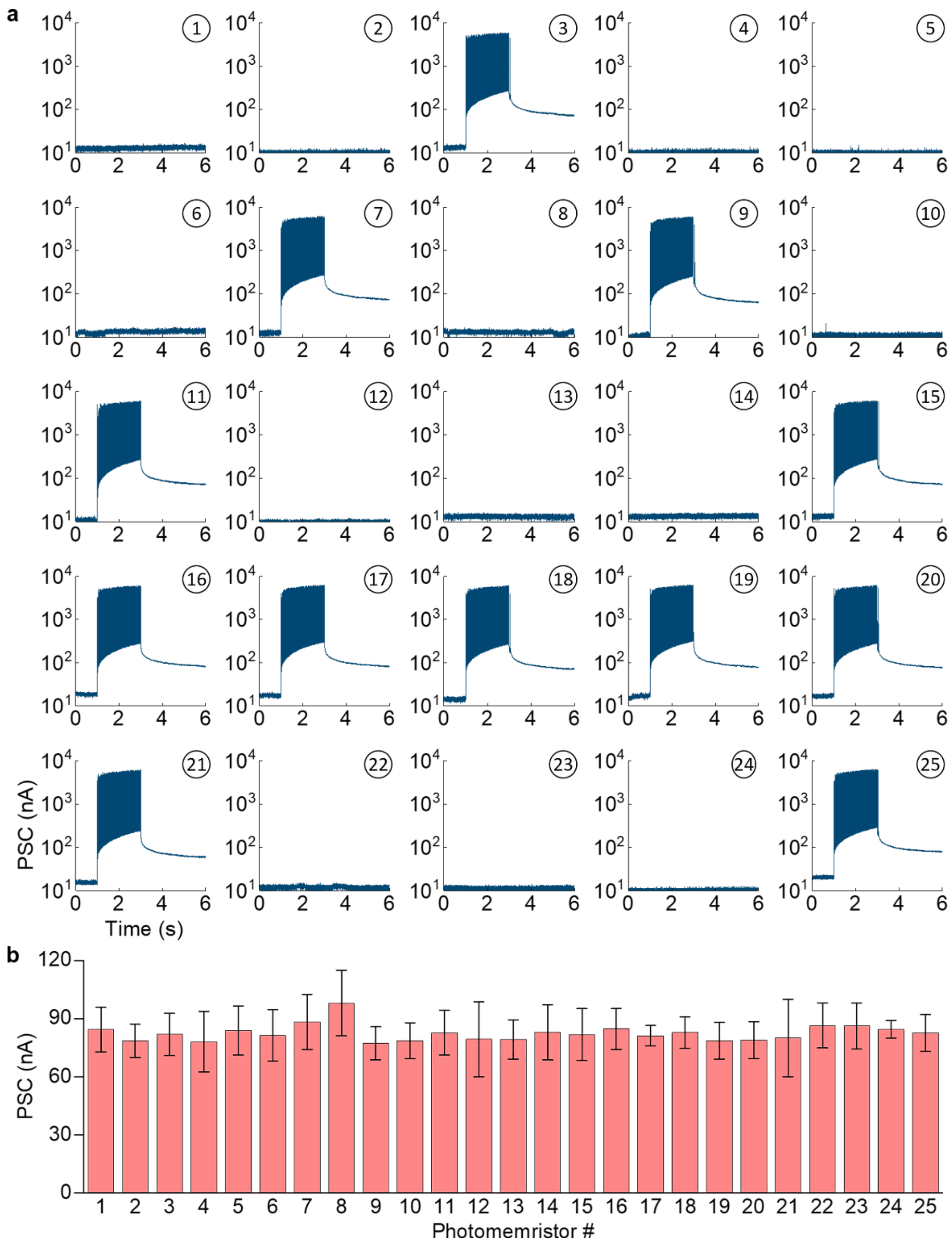

Supplementary Figure 4. PSC response and statistical analysis of photomemristors. a, PSC response when ‘seeing’ the image ‘A’. The PSC response is triggered by the projection of an optical image of the letter ‘A’ onto a 5 × 5 array of photodetectors for 2 s (from $t = 1$ s to $t = 3$ s). The spiking rate of the PSC signal during illumination does not depend on bias voltage and can be used for detection. The PSC value after optical input depends on the bias voltage and acts as attention-dependent vision memory. The data

in this figure were obtained with a bias of 2 V. The optical image of the letter ‘A’ was produced by a blue LED and a shadow mask. **b**, Photomemristor-to-photomemristor and cycle-to-cycle variation. The PSC values of the photomemristors were recorded at t = 6 s (3 s after optical illumination). The red bars indicate the mean PSC values of the 25 photomemristors and the error bars indicate standard variations obtained from the responding photomemristors during the 26 measurements of the visual memory of A-Z. The device-to-device variation of PSC values in the photomemristors does not affect the tactile-to-visual recognition in our experiments, as the memorized vision is represented by binary images and the recognized vision is represented by normalized vectors. For applications that use analog PSC values, the effect of device-to-device variation and noise could be minimized by a using step function to pre-process the PSC signals.

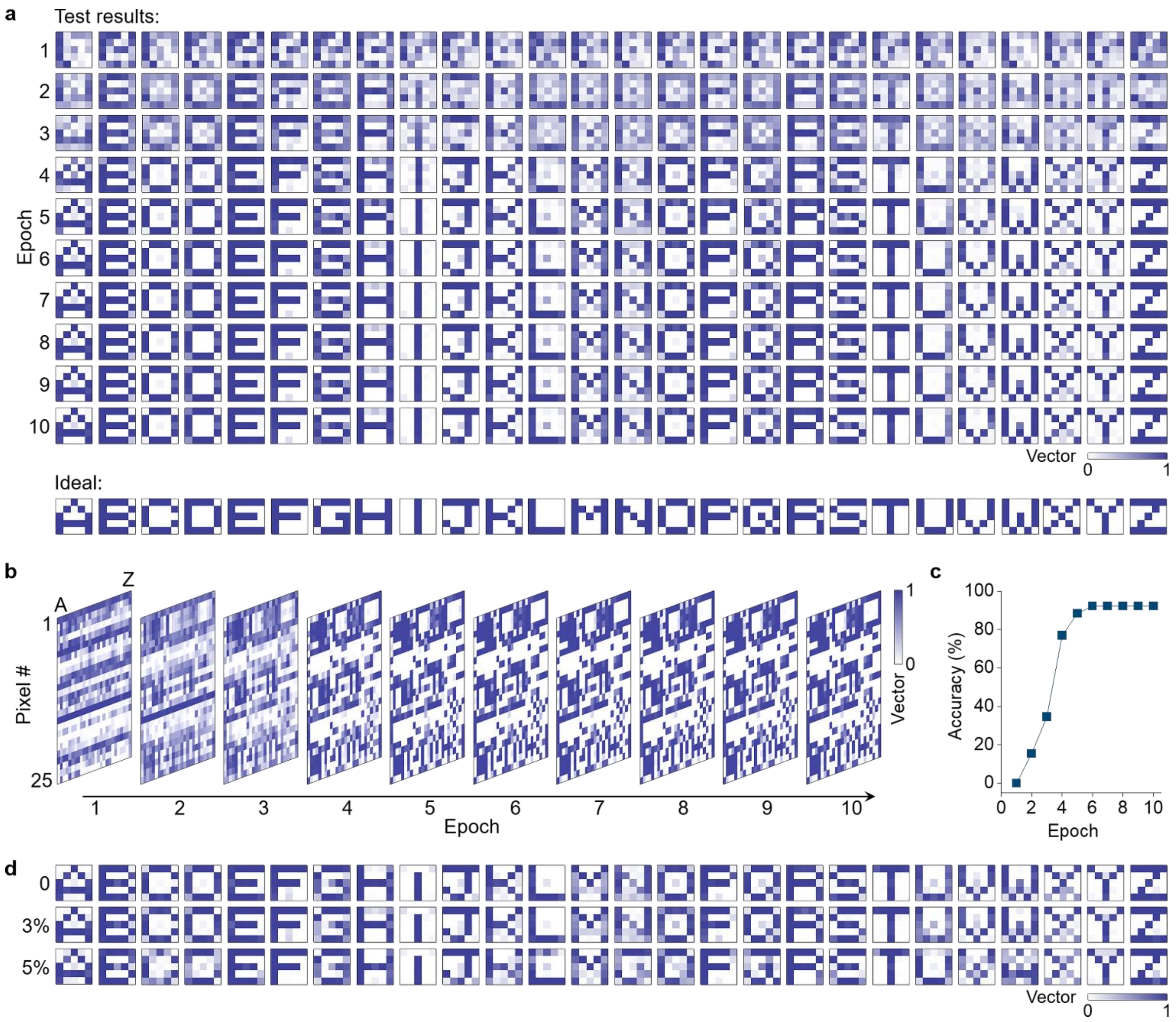

Supplementary Figure 5. Test results of the tactile-vision system. a, Reproduced alphabet letters by tactile inputs during 10 training epochs. With the number of epochs increasing, the visualized alphabet letters become clearer. After 10 training epochs, only the letters ‘C’ and ‘D’ are recognized more like an ‘O’. This small inaccuracy is explained by the similar handwriting process of ‘C’, ‘D’ and ‘O’. The bottom row shows the ideal images of the alphabet letters. **b**, Library of reproduced vision vectors shown in (a), as a function of the number of training epochs. **c**, Recognition accuracy of alphabet letters upon handwritten input as a function of the number of training epochs (data derived from (a) and (b)). After 6

epochs, the tactile-visual MSeNN system visualizes the tactile input with an accuracy of 92%. **d**, Test results for 3% and 5% added noise to the tactile vector inputs. The recognition accuracy decreases from 92.3% (no noise) to 88.5% (3% noise) and 61.5% (5% noise).

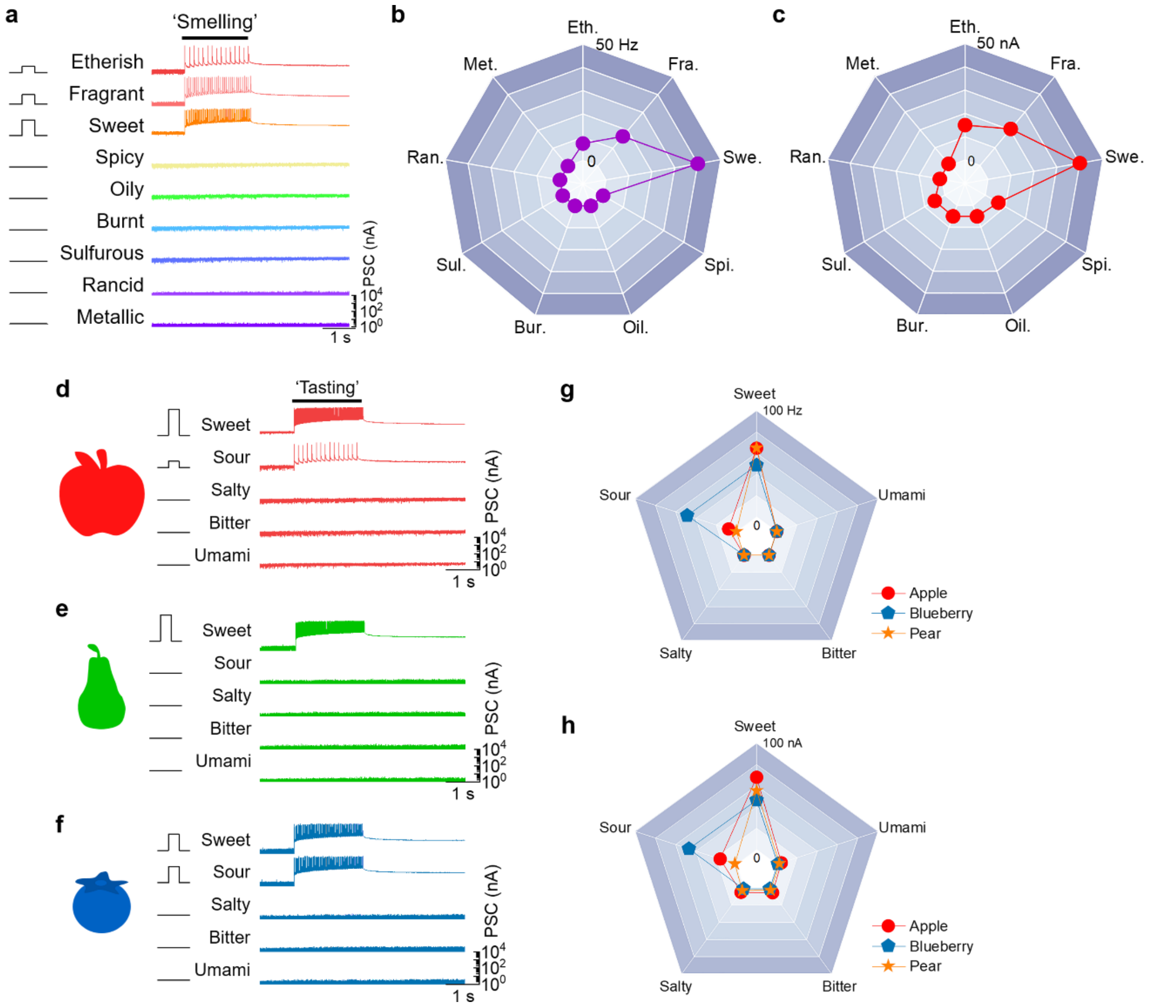

Supplementary Figure 6. Artificial olfactory and gustatory systems. a, PSC signals produced by the nine photomemristors of the artificial olfactory system with simulated inputs representing the smell of a fruit that is sweet and a little fragrant and etherish. **b**,**c**, PSC spiking rate and PSC value of the artificial olfactory system (vector plots derived from data in (a)). The 9-dimensional features in (b) and (c) indicate sensing and memorizing of smell information. **d-f**, PSC signals produced by the 5 photomemristors of the artificial gustatory system with simulated inputs representing the taste of apple (d), pear (e), and blueberry (f). **g**,**h**, PSC spiking rate and PSC value of the artificial gustatory system (vector plots derived from (d-f)). The 5-dimensional features in (g) and (h) indicate sensing and memorizing of taste information.

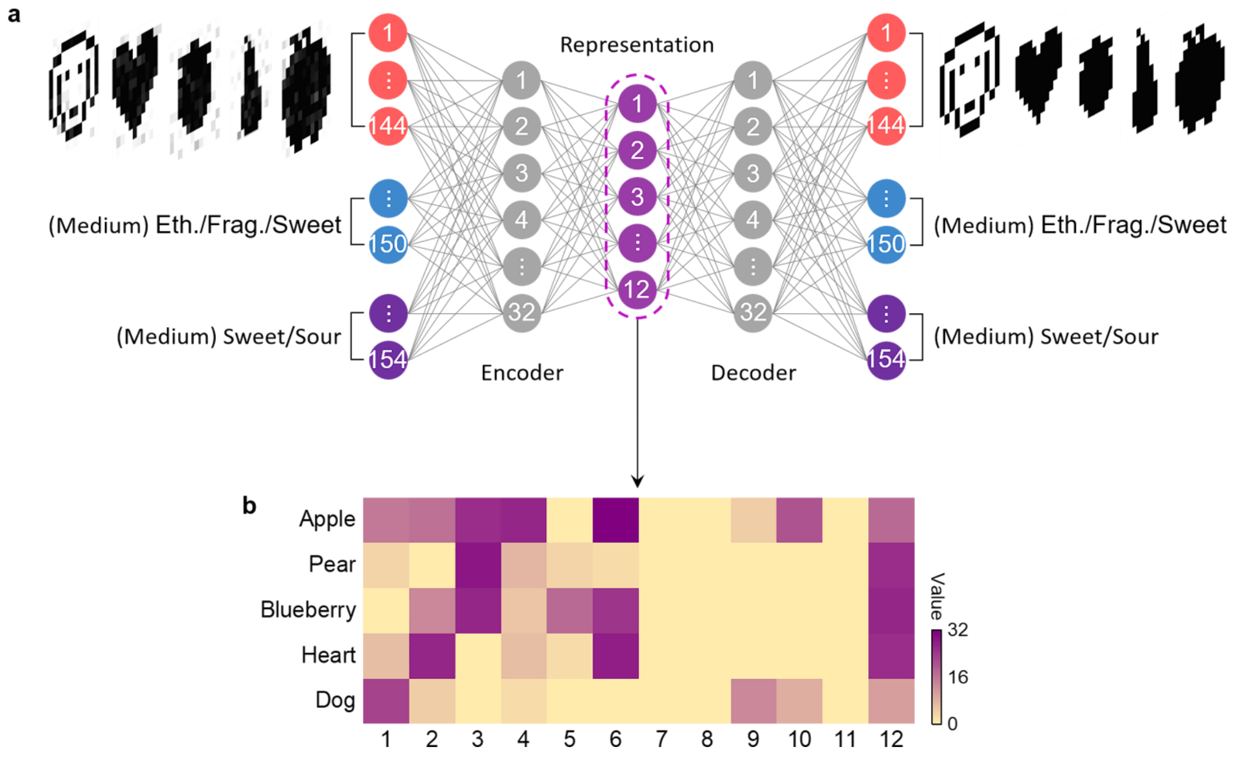

Supplementary Figure 7. Autoencoder. a, Schematic structure of the autoencoder. The autoencoder automatically encodes image, smell, and taste information into a 12-dimensional representation. The input and output are binary 0/1. The images have 12 × 12 pixels, corresponding to 144-dimensional vector inputs. The 6-dimensional smell vectors represent etherish, fragrant, sweet, medium etherish, medium fragrant, and medium sweet. The 4-dimensional taste vectors represent sweet, sour, medium sweet, and medium sour. **b**, Learned representation of the image/smell/taste information of an apple, pear, blueberry, heart, and dog. The representation is learned in an unsupervised manner. The learned representation supervises the training of the ANN with audio input (see Figure 5b). More details on the autoencoder are given in the Methods section.

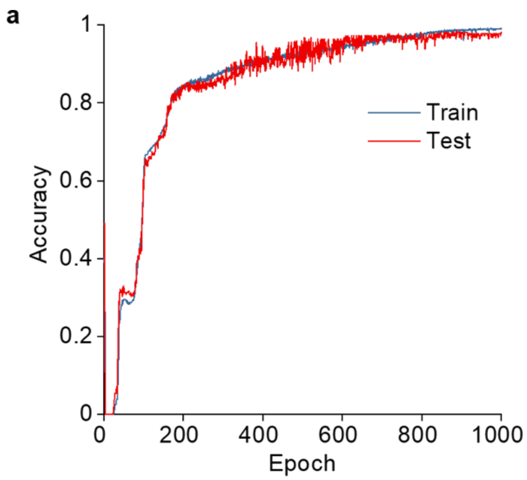

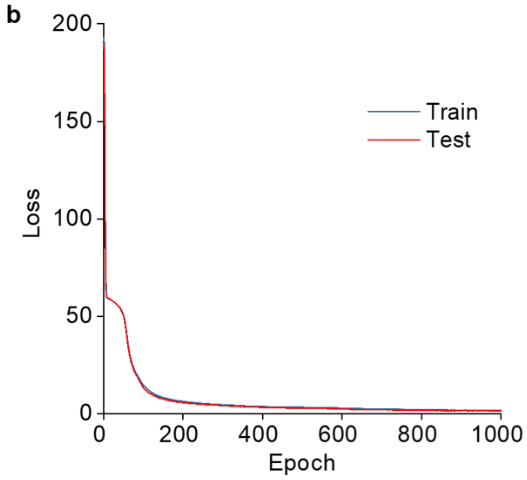

Supplementary Figure 8. Training and testing of the auditory-vision/olfactory/gustatory system. Accuracy (a) and loss (b) of the system during training and testing. Training is performed with 1980 data sets and testing is conducted with 220 data sets. Accuracy is defined as the recognition rate of the 12-dimensional representations learned by the autoencoder in each epoch. Loss is defined by the mean square error function (see Supplementary Note 1).

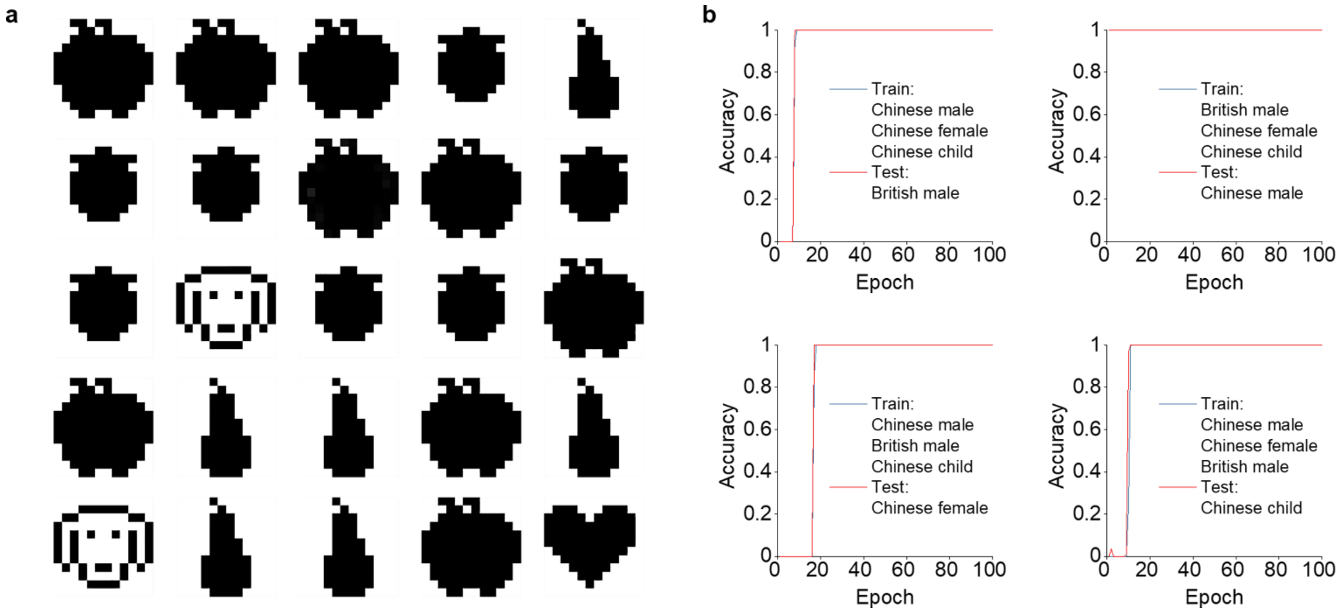

Supplementary Figure 9. Training and testing of the auditory-vision system. a, 25 sets of test results of audio-visual recognition. As input, words spoken with different accents (fruits) and barking by a Labrador Retriever and Cocker Spaniel (dog) are randomly used. **b**, Accuracy of training and testing when different accents (British male, Chinese male, Chinese female, Chinese child) are used to pronounce the word 'apple' during training and testing. High recognition accuracy is attained irrespective of the accent.

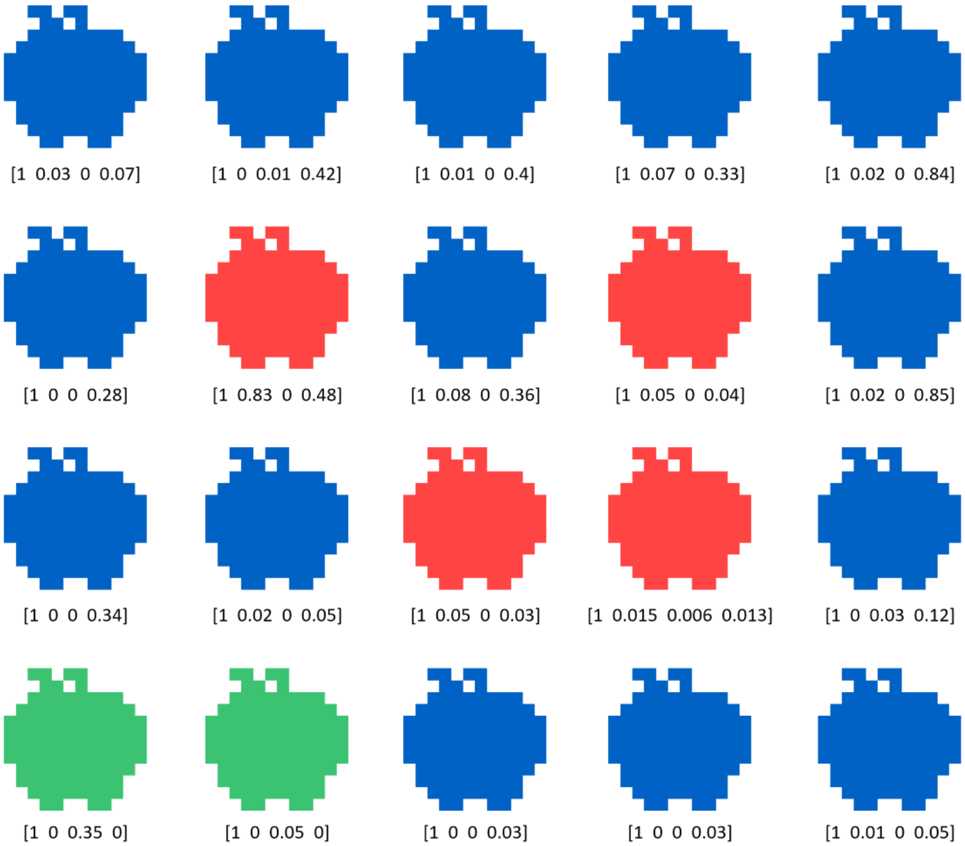

Supplementary Figure 10. Crossmodal imagination. 20 predicted images obtained when /bluː, ˈapəl/ is given as the audio input during testing. The ANN of the auditory-vision system was trained by audio inputs (red, green, blue, red apple, green apple) under the supervision of 4-dimensional representation vectors. The elements of the representation vectors indicate apple, red, green, and blue. In the representation, the color with the highest value is considered as the predicted color of the apple. The results indicate 70% accuracy in predicting the image of a blue apple that was never heard or seen during training.

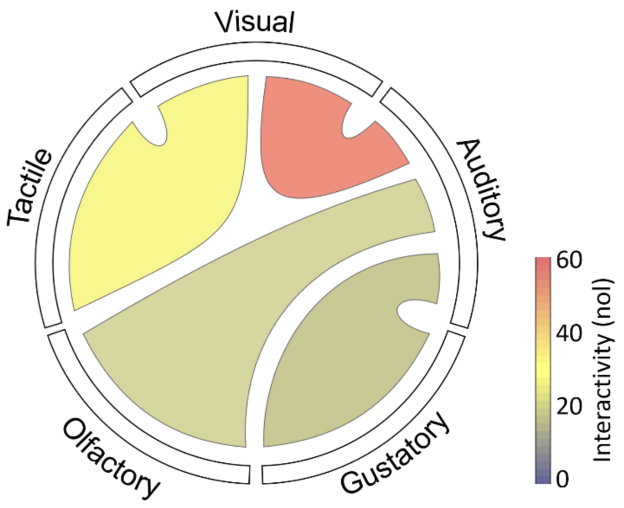

Supplementary Figure 11. Interactivity of the MSeNN. In the artificial MSeNN with multisensory fusion, the five senses interact with each other via an ANN. To describe how much information the senses integrate, we define an interactivity parameter. The interactivity has a unit of *nol*, corresponding to the *number of letters* of the integrated information. Without any sensory information detected in a fresh system, the map is blank, indicating a 'newborn' MSeNN system. With more and more sensory information detected and integrated, the MSeNN system keeps learning and updating its interactivity map. The colors of the map represent a 'knowledgeable' state. In this graphical representation, the edge areas of the circle indicate the five senses – vision, touch, hearing, smell, and taste – and the four parts inside the circle connecting two senses indicate the interactivity between visual and tactile/auditory/olfactory/gustatory information. The color scale visualizes the interactivity in *nol*. For example, in this work, vision and touch interact with each other, and the integrated information are the letters of the alphabet A-Z (interactivity of 26 *nol*). Similarly, the interactivities between hearing and vision/smell/taste are 56/18/18 *nol*. The interactivity map thus summarizes the integrated sensory information in this study, wherein we mostly focus on the integration of vision with the other four primary senses. The concept of multisensory neural network integration presented here extends easily to other sensory interactions.

Supplementary Table 1. Summary of recent typical artificial multisensory systems.

Systems	Sensors	Communication	Coding	Synaptic processing	Integrated information	Recognize	Imagine	Ref.
BSV	Somatosensory	DC	Amplitude	_	Somatosensory-visual	Gesture	_	[10]
BASE	Touch<br>Vision	DC	Amplitude	Dendritic integration	Visual-haptic	Multi-transparency pattern	_	[11]
**MSeNN**	**Touch<br>Vision<br>Hearing<br>Simulated smell<br>Simulated taste**	**Optical spikes**	**Rate coding<br>Temporal coding**	**Decoding<br>Filtering<br>Memory<br>Crossmodal integration**	**Visual-tactile-auditory-(simulated) smell/taste**	**Multiple crossmodal information<br>Simple overall semantic recognition**	**Blue apple**	**This work**

Supplementary References

1. King, A. J. Multisensory integration. *Science* **261**, 928-929 (1993).
2. McDonald, J. J., Teder-Sälejärvi, W. A. & Ward, L. M. Multisensory integration and crossmodal attention effects in the human brain. *Science* **292**, 1791 (2001).
3. Stein, B. E., Stanford, T. R. & Rowland, B. A. Development of multisensory integration from the perspective of the individual neuron. *Nat. Rev. Neurosci.* **15**, 520-535 (2014).
4. Talsma, D. Predictive coding and multisensory integration: an attentional account of the multisensory mind. *Front. Integr. Neurosci.* **9**, 19 (2015).
5. Tang, X., Wu, J. & Shen, Y. The interactions of multisensory integration with endogenous and exogenous attention. *Neurosci. Biobehav. Rev.* **61**, 208-224 (2016).
6. Kato, H. & Harada, T. Image reconstruction from bag-of-visual-words. IEEE Conference on Computer Vision and Pattern Recognition 955-962, Ohio, U. S. 24 June 2014.
7. Tan, H. et al. Tactile sensory coding and learning with bioinspired spiking afferent nerves. *Nat Commun.* **11**, 1369 (2020).
8. Kim, Y. et al. A bioinspired flexible organic artificial afferent nerve. *Science* **360**, 998-1003 (2018).
9. Tee, B. C.-K. et al. A skin-inspired organic digital mechanoreceptor. *Science* **350**, 313-316 (2015).
10. Wang, M. et al. Gesture recognition using a bioinspired learning architecture that integrates visual data with somatosensory data from stretchable sensors. *Nat. Electron.* **3**, 563–570 (2020).
11. Wan, C. et al. An artificial sensory neuron with visual-haptic fision. *Nat. Commun.* **11**, 4602 (2020).
